# Improved Hydrophobicity of Macroalgae Biopolymer Film Incorporated with Kenaf Derived CNF Using Silane Coupling Agent

**DOI:** 10.3390/molecules26082254

**Published:** 2021-04-13

**Authors:** Adeleke A. Oyekanmi, N. I. Saharudin, Che Mohamad Hazwan, Abdul Khalil H. P. S., Niyi G. Olaiya, Che K. Abdullah, Tata Alfatah, Deepu A. Gopakumar, Daniel Pasquini

**Affiliations:** 1 School of Industrial Technology, University Sains Malaysia, Penang 11800, Malaysia; abdulkan2000@yahoo.com (A.A.O.); ngolaiya@futa.edu.ng (N.G.O.); ck_abdullah@usm.my (C.K.A.); tataalfatah83@gmail.com (T.A.); deepu1789@gmail.com (D.A.G.); 2 Chemistry Institute, Federal University of Uberlandia-UFU, Uberlândia 38400-902, Brazil; danielpasquini2013@gmail.com

**Keywords:** macroalgae, kenaf fibre, silane treatment, hydrophilicity, nanocellulose, reinforcement

## Abstract

Hydrophilic behaviour of carrageenan macroalgae biopolymer, due to hydroxyl groups, has limited its applications, especially for packaging. In this study, macroalgae were reinforced with cellulose nanofibrils (CNFs) isolated from kenaf bast fibres. The macroalgae CNF film was after that treated with silane for hydrophobicity enhancement. The wettability and functional properties of unmodified macroalgae CNF films were compared with silane-modified macroalgae CNF films. Characterisation of the unmodified and modified biopolymers films was investigated. The atomic force microscope (AFM), SEM morphology, tensile properties, water contact angle, and thermal behaviour of the biofilms showed that the incorporation of Kenaf bast CNF remarkably increased the strength, moisture resistance, and thermal stability of the macroalgae biopolymer films. Moreover, the films’ modification using a silane coupling agent further enhanced the strength and thermal stability of the films apart from improved water-resistance of the biopolymer films compared to unmodified films. The morphology and AFM showed good interfacial interaction of the components of the biopolymer films. The modified biopolymer films exhibited significantly improved hydrophobic properties compared to the unmodified films due to the enhanced dispersion resulting from the silane treatment. The improved biopolymer films can potentially be utilised as packaging materials.

## 1. Introduction

The biopolymers have been considered for a wide range of applications as a replacement for synthetic polymers, which constitute environmental problems resulting from their poor degradability properties. Biopolymers have improved environmental performance than synthetic polymers [1]. Consequently, biopolymers have a comparative advantage over synthetic polymers due to their intrinsic properties, including their biocompatibility, biodegradability, and renewability potentials [2]. In recent years, macroalgae-derived polymers have gained remarkable attention as a functional material compared to land-based biomass due to the predominance of cellulose and little fractions of hemicellulose lignin [3].

Macroalgae such as agar, alginate, and carrageenan have been proposed as potential packaging materials. Macroalgae biomass has promising prospects due to its inherent properties of a low content of natural physicochemical barriers and higher yields of pure cellulose than other land-based biomasses [4]. Furthermore, the extraction of nanocellulose from cellulosic macroalgae composition can potentially be utilised to reinforce biodegradable polymers. Macroalgae-based films can exhibit desirable properties, especially for packaging films application [5,6]. Many researchers had reported the potential application of macroalgae for the development of biopolymer films [7,8]. Carrageenan is a water-soluble polysaccharide that is derived from red algae. Its surface functionality is suitable for many applications. Carrageenan has good film-forming properties that can be attributed to its considerable amount of sulphonic groups in its structure, which allows the formation of the film through the self-aggregation of its helical structures [9]. However, these films’ hydrophilic behaviour attributed to poor moisture barrier properties and low water resistance has become a major challenge among polymer scientists. The reinforcement of the films to ensure enhanced properties using nanocellulose is very significant.

Cellulose nanofibrils (CNFs) have many better-reinforcing potentials as a replacement for petrochemical-based materials [10,11]. CNFs have been explored to improve the moisture resistance, barrier, and mechanical properties of biopolymers derived for various applications such as packaging, scaffolds in tissue engineering, and biomedical. CNF has been explored as a building block to produce high-performance bio-materials [12]. The utilisation of CNF from agricultural-based precursors is attributed to their abundance, biodegradability, and ease of fibre extraction, including their suitability for interfacial bonding in polymer composites [13,14]. Kenaf bast fibre is a non-woody lignocellulosic material that is a very suitable precursor for the extraction of CNF [15]. Kenaf bast fibre contains an abundance of cellulose content and low specific gravity [16]. Kenaf bast fibres have superior properties as reinforcement agents [17]. The fibre’s high specific strength and modulus have attracted significant interests due to the good mechanical properties, large specific surface area, and low coefficient of thermal expansion, including high aspect ratio [18].

Previous studies have incorporated Kenaf-based CNF into the matrix of films as reinforcement to improve biopolymer’s mechanical properties. The potentials of isolated CNF from Kenaf fibre have been reported as reinforcement in polylactic acid [19], composite [20], starch-based composite film [21,22]. However, macroalgae/Kenaf-derived CNF film’s hydrophilic behaviour is a significant drawback due to its poor moisture resistance behaviour. Therefore, there is a need for modification to improve its surface hydrophobicity. To the best of our knowledge, no studies have reported the surface modification of macroalgae/CNF film using silane coupling agent to improve the interfacial chemical bonding and the hydrophobicity of the biopolymer. Silane interfacial coupling agent is adopted for this work due to its potential of forming stable covalent bonding to the biofilm surface. In view of the inherent functional characteristics of silane based on its water-repellent and adhesion properties, surface modification and, subsequently, silane treatment were applied to improve the surface properties of the macroalgae/CNF-derived biopolymer.

## 2. Materials and Method

### 2.1. Materials

Kenaf plants were obtained from Nibong Tebal Seberang Perai, Pulau Pinang, Malaysia. CNF was isolated from Kenaf bast and used as nanofibre reinforcement in the matrix. The removal of bast fibres from its stems was achieved using a Sprout Bauer refiner (USA) model number R34EM, Fitchburg, MA, USA. SC-CO_2_ was purchased from ZARM scientific and supplies Bukit Mertajam, Penang, Malaysia (Malaysia). In the present work, Union Carbide Ltd. Sheffield, UK supplied a stock solution of silane (SILRES BS 1701). All chemicals, such as sodium hydroxide (NaOH), sodium chlorite (NaClO_2_), hydrogen peroxide (H_2_O_2_), acetic acid (CH_3_COOH), were supplied by Sigma-Aldrich, Subang Jaya, Selangor, Malaysia and were of technical grade.

### 2.2. Isolation and Characterisation of CNF via Supercritical CO_2_

About 500 g oven-dried weight raw kenaf bast fibres were heated at 160 °C for 3.5 h in an alkaline medium consisting of 25 wt% of NaOH and 0.2 wt% of anthraquinone (AQ) to obtain alkali-treated fibres, which were washed with deionised water to remove surface impurities and also to remove degraded hemicellulose including other extractives. After that, a bleaching treatment was conducted using 3% H_2_O_2_ and 0.5% MgSO_4_ at 80 °C for 2 h. The bleached fibres were subjected to SC-CO_2_ explosion at 60 °C for 2 h to produce SC-CO_2_ CNF. In the final stage, mild acid hydrolysis using 5% oxalic acid was added to the SC-CO_2_ CNFs to avoid the toxicity of the produced cellulose nanofibres CNFs. The SC-CO_2_-extracted CNF was used as a reinforcement material in macroalgae biofilm. The isolated CNFs were characterised using TEM, particle size, and zeta potential. TEM was used to investigate the nanostructure of CNF, which was investigated using TEM Philips model CM300-FEG at an operational acceleration voltage of 100 kV. The zeta potential of the CNFs was examined using a DLS Malvern Zetasizer 7.11. Distilled water was added to the dispersant, after which samples were sonicated for 10 min. The set parameters used to obtain the measurement include viscosity, 0.8872, and the refractive index of distilled water and material: 1.330 and 1.47, respectively. The adjustment of pH was achieved using NaOH and HCl. The zeta potential measurement was obtained as a function of an aqueous suspension of CNF in a 0.1 mM KCl electrolyte.

### 2.3. Preparation of Macroalgae/CNF Biopolymer Film

About 4 g of oven-dried raw macroalgae and 2 g of glycerol were dissolved in 200 mL of deionised water and then heated at 85 °C with constant stirring to obtain a suspension. Glycerol was used in the study as the plasticiser agent to the macroalgae/CNF biopolymer films. Glycerol was used as a hydrophilic dispersant for CNF and served as a hydrophilic plasticiser for macroalgae. The percentage concentration of fibres incorporated in the macroalgae biopolymer films were 1%, 2%, 3%, 4%, and 5%. Finally, dried macroalgae biopolymer was obtained at 45 °C for 24 h after casting the hot solution on the plate. Before investigating and analysing test samples, the oven-dried macroalgae biopolymer films were peeled off from the plate. They were put in the desiccator at a relative humidity of 50% for 2 days.

### 2.4. Surface Treatment of Macroalgae Film Using Silane

Mercerization and surface functionalisation of macroalgae/CNF biopolymer films was adopted from [23] with modifications. To improve the prepared macroalgae/CNF film’s surface hydrophobicity, silane treatment was conducted on the film as a hydrophobic coupling agent. The films were soaked in 5% diluted silane in methanol. The chemical modification process took place in a fume chamber using triethoxymethylsilane. The surface modification of the biopolymer films was achieved using acetone mixed with a 40% silane solution. Macroalgae/CNF biopolymer films were put in the reaction flask, including the prepared solution. A reaction flask was attached to the reflux condenser with constant water flow to activate the condensation process. The reaction flasks with a reflux condenser on top were soaked in silicone oil, and the system was properly set up to avoid error during the modification process. The modifications process was run for 5 h at 70 °C. Once the modifications were achieved, the macroalgae/GL-CNF biopolymer films were placed and cleaned in beakers using acetone to remove the excessive silane. The films obtained were later vacuum dried at 70 °C for 3 h. Finally, the films were oven-dried at 40 °C for 24 h.

### 2.5. Characterisation of Macroalgae Biopolymer Films

The tensile, morphology, structural, wettability, and thermal properties of the biofilms were investigated using tensile test, SEM, FT-IR, contact angle, and thermogravimetric analysis/derivative thermal gravimetry (TGA-DTG) analysis. The tensile tests were investigated according to the standard method; the morphology characterisation was conducted using scanning electron microscopy SEM Leo Supra 50 VP Field Emission (Carl-Zeiss SMT, Oberkochen, Germany). The films’ surface functional properties were investigated using FT-IR Spectrum 8900 IR Spectrometer (Shimadzu, Chiyoda-ku, Tokyo, Japan). The contact angle measurements were evaluated using KSVCAM 10 (KSV Instruments Ltd., Espoo, Finland). The thermal properties were investigated using Mettler-Toledo thermogravimetric analyser model TGA, Schwarzenbach, Switzerland. The tensile properties of the films were conducted to investigate the strength of the biofilm, and SEM was used to analyse the fracture surface. The active functional groups on the biopolymer films were investigated using the FT-IR. The biopolymer films’ wettability was investigated, and contact angle experiments were conducted to determine the surface behaviour of the biofilm with distilled water. The evaluation of biofilm thermal stability was achieved using the thermogravimetric analysis (TGA).

The tensile strength, tensile modulus, elongation at break, and toughness were investigated using the universal testing machine, model 5900 Series, Instron Engineering Co Norwood, MA, USA. The tensile strength was examined according to ASTM D882-02. Samples with dimensions of 30 mm length and 5 mm width were placed within the testing machine’s grip heads. The cross-head speed and the length of the initial gauge were 50 mm/min and 50 mm, respectively. Five test specimens were conducted for each sample at a varying concentration of 1%, 2%, 3%, 4%, and 5%. The samples were evaluated at a speed of 1 mm/min. The macroalgae/CNF biopolymer film’s fractured morphology was investigated using a scanning electron microscope, SEM (S-3400N, Hitachi, Japan). Before the observation, samples were cut into 10 mm × 10 mm and oven-dried at 60 °C and were gold-coated to enhance samples’ conductivity. The images were captured at an operational range of 5–20 kV.

The biopolymer films’ functional group analysis was examined using the Fourier Transforms Infrared Spectrophotometer (FT-IR). The FT-IR spectra were obtained within the range of 4000–600 cm^−1^ using FT-IR Nicolet, Thermo Scientific Inc iS10, Madison, WI, USA.

The biopolymer films’ contact angle surface property was determined using a goniometer model optical contact angle (OCA) 15EC, Filderstadt, Germany. The biofilms were cut into 1 × 1 mm^2^ dimension. The films were placed on a flat edge and attached to the CA analyser. Measurements were observed in triplicate. The thermal properties of the macroalgae CNF biopolymer films were evaluated using a Mettler-Toledo thermogravimetric analyser model (Mettler Toledo, Schwarzenbach, Switzerland) in the range of 50 to 800 °C at a heating rate of 20 °C/min to obtain the thermal gravimetric analysis (TGA) and derivative thermal gravimetry (DTG) curves.

The surface morphology and topography of the macroalgae CNF biopolymer films were analysed using the atomic force microscope (AFM) manufactured by NanoFocus Inc, Guro Gu, Seoul, Korea. Experiments were performed in an ambient air environment. Scanning was achieved at a scan speed of 1 Hz. The biofilm surface’s topographical images were obtained using the WSx M image viewer, and the surface roughness values obtained.

## 3. Results and Discussion

### 3.1. Characterisation of Macroalgae/CNF Biopolymer Film

**Figure 1:**
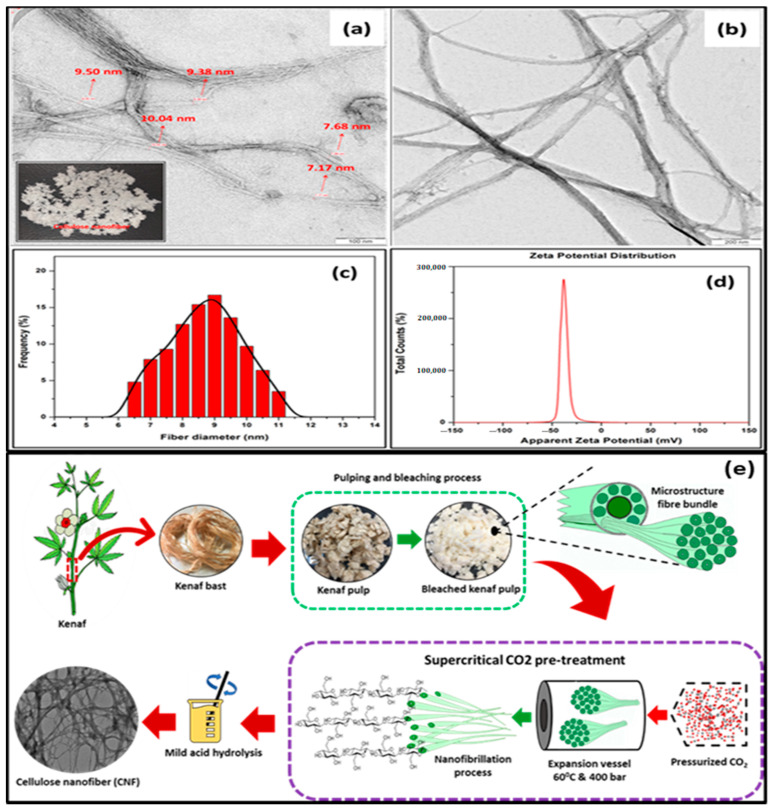
TEM micrograph of a cellulose nanofibril (CNF) (**a**) 10,000× magnification, (**b**) 40,000× magnification, (**c**) particle size distribution of CNF, and (**d**) zeta potential distribution of CNFs (**e**) Schematic diagram of CNF preparation.

The morphological properties of CNF isolated from Kenaf bast fibres were analysed using TEM at 10,000× magnification (Figure 1a) and 40,000× magnification (Figure 1b). The particle size and potential zeta distribution were represented in Figure 1c,d, respectively. The schematic diagram showing the step-by-step procedure for CNF isolation from Kenaf bast fibre is presented in Figure 1e. The surface structure of the Kenaf-based CNFs indicated no distortion in the amorphous region of the isolated CNFs in Figure 1a, which signifies its potential for reinforcement even though the reduction in the thickness of the fibres was observed in Figure 1b. This may be attributed to the changes observed in the colour of suspension when heated during the bleaching of fibre [18]. The effect of bleaching may have reduced the thickness of the fibre diameter due to the decomposition of the lignocellulosic content of the fibre.

This implies that the SCO_2_ assisted chemical pre-treatment and the impact of high pressure disrupted secondary interaction of fibres due to defibrillation, which resulted in the effectiveness of the extraction of CNFs from bast fibres. The effect of pre-treatment induces repulsive charges on the fibres influencing their subsequent defibrillation process [24]. The diameter of fibres was evaluated to be around 7.17–9.50 nm using image J software. The data were correlated with the diameter of the fibres distribution in Figure 1c. The findings revealed that Kenaf bast fibre-based CNF exhibited an average diameter of 6.5 nm, with about 4.8% distribution achieved. An observable percentage fibre distribution trend increased with increasing diameter of fibres until the highest frequency of fibre distribution was recorded at approximately 19%; this was achieved at 9 nm average diameter of CNF. A decrease in percentage in the frequency of fibres distribution was observed as the diameter of the fibre increased [25]. Figure 1c illustrates the value of the zeta potential of the SCO_2_-extracted CNF suspension under mild acid hydrolytic conditions. A noticeably high negative value of −31.0 ± 4.1 mV was achieved. The high negative zeta potential of the extracted CNFs could be attributed to the oxalate group’s presence on the surface, which was formed during the hydrolysis of oxalic acid. The high value of zeta obtained indicated stable suspension of CNFs due to the absolute value achieved, which was higher than −25mV. This value suggested that colloidal stability was attained due to sufficient repulsive force required [26].

### 3.2. Morphology of the Unmodified and Modified Macroalgae/CNF Biopolymer Film

**Figure 2:**
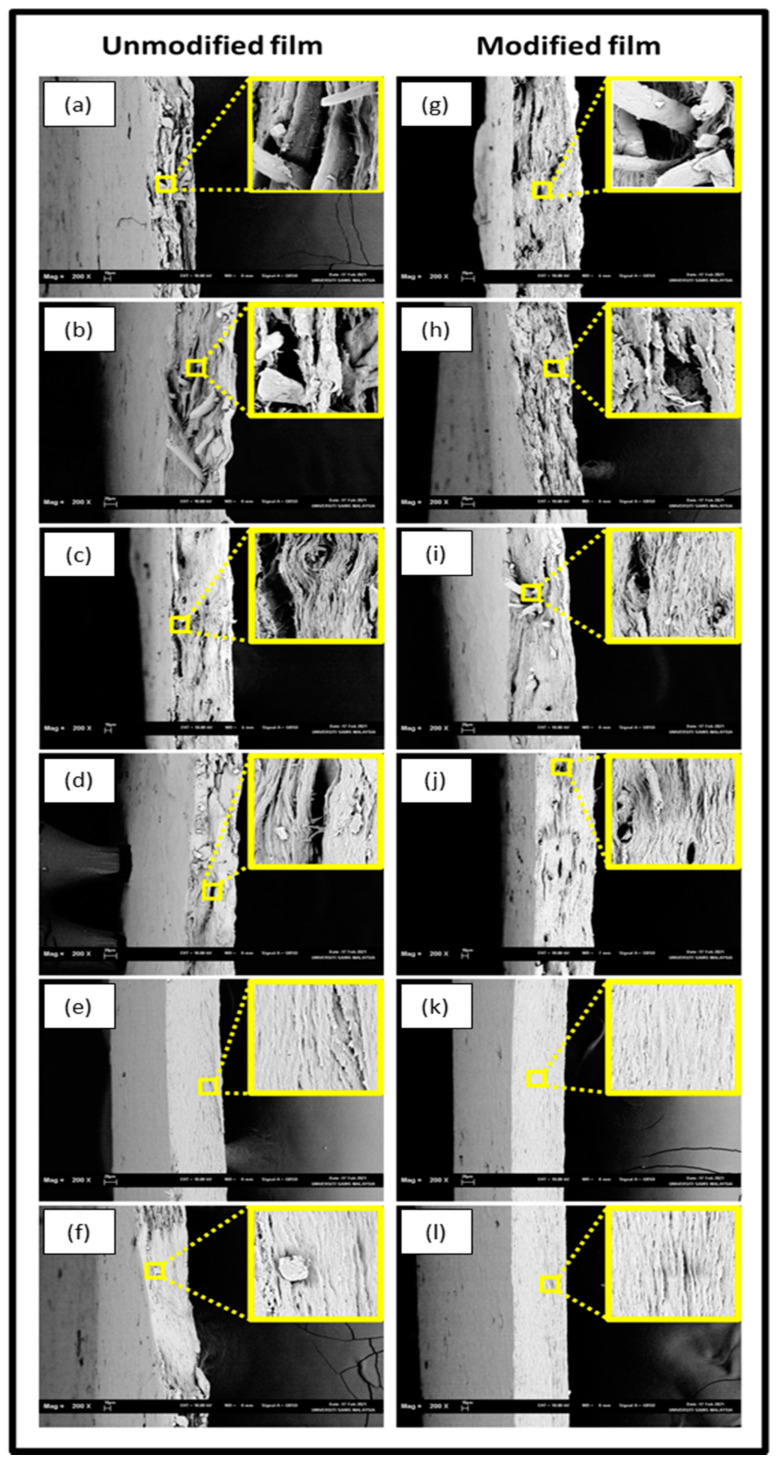
Fracture morphology of (**a**) control macroalgae film, (**g**) control unmodified macroalgae CNF film with fibre loading of (**b**) 1%; (**c**) 2%; (**d**) 3%; (**e**) 4%; (**f**) 5%; and modified CNF film with fibre loading of (**h**) 1%; (**i**) 2%; (**j**) 3%; (**k**) 4%; and (**l**) 5%.

The SEM morphology of the CNF-reinforced biopolymer film at different loading for modified and unmodified is presented in Figure 2. The unmodified biopolymer films are shown in Figure 2a–f, and the modified macroalgae/CNF biopolymer films are illustrated in Figure 2g–l. The controlled films for unmodified and modified were represented at 0% fibre loading in Figure 2a,g, respectively. Both films indicated a porous surface that better explained the need for enhancement to improve the biopolymer films’ mechanical properties. However, the significance of incorporating CNFs resulted in the reduction of the porous surface of the biopolymer films for both modified and unmodified, which was noticed as the fibre loading increased. However, the modified biofilms in Figure 2g–f exhibited dense structure with tightly packed stratified cross-sections as the fibre loading increased compared with unmodified biofilms. As the percent loading increased, the biofilm’s porosity decreased, suggesting the significance of homogenous and well dispersed CNFs in the macroalgae matrix, which was fused due to the interaction and intermolecular hydrogen bonding [27]. The reduction of the biopolymer film’s porous surface could be attributed to the increase in CNFs in the macroalgae matrix, indicating an increase in mechanical strength. Beyond, 4% fibre increase, agglomeration of CNF in the voids were observed, resulting in a decrease in the mechanical properties. The CNFs nanoparticles were observed to fill up voids in the macroalgae structure, resulting in a compacted film. The effect of agglomeration results in defects in the interfacial region; thus, it reduces the fibre-matrix interaction.

### 3.3. Mechanical Properties of Macroalgae/CNF Biofilm

**Figure 3:**
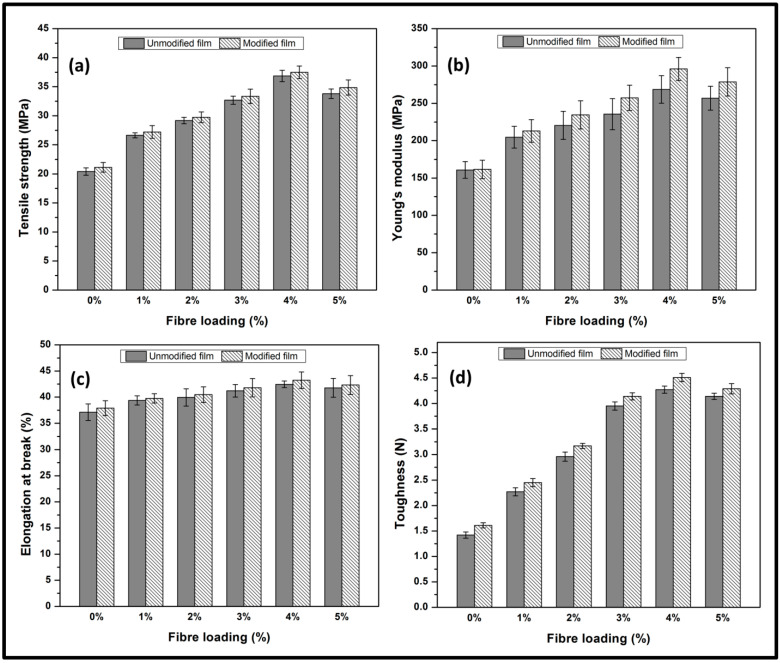
Mechanical properties of unmodified/modified macroalgae film with CNF loading, (**a**) tensile strength, (**b**) Young modulus, (**c**) elongation at break, and (**d**) toughness.

The tensile properties of the macroalgae/GL-CNF-based Kenaf bast fibre biopolymer film are illustrated in Figure 3. The modified biopolymer films’ tensile strength tends to increase compared to the unmodified films as the percent fibre loading was increased up to about 37.5 MPa. Although a decrease in tensile strength was achieved at 5% fibre loading, the modified biopolymer films still recorded higher tensile strength than the unmodified biopolymer films. This is indicative that the silane chemical properties impacted the compatibility between the CNF and the macroalgae matrix. By extension, it enhanced the tensile strength of the biopolymer film. Generally, the improved tensile strength could be attributed to the good dispersion of CNF in the macroalgae matrix, as indicated in the SEM images. Much more, the probable formation of intermolecular hydrogen bonding of the hydroxyl group in the Kenaf bast fibre CNF and the macroalgae matrix significantly enhanced the film’s tensile strength.

Furthermore, an increase in the tensile modulus was achieved as the percent fibre loading was increased for both modified and unmodified film. In particular, the highest modulus was achieved at 4% fibre loading for both modified and unmodified film. The incorporation of CNF in the macroalgae matrix improved the tensile strength and modulus. The increase in the tensile strength and modulus indicates good dispersion of CNFs within the polymer matrix, which influenced adhesive interaction of the CNFs reinforcement in the macroalgae matrixes [28]. When compared to unreinforced macroalgae films, enhancement in the tensile modulus was due to the uniform dispersibility and compatibility of CNF fibres in the macroalgae matrices. This better dispersion and compatibility resulted in the efficient and uniform load transfer from the macroalgae matrixes to the CNF networks. The increased mechanical strength of the macroalgae films after incorporating CNFs may be attributed to the increased stiffness of the films [12]. It also revealed the high reinforcing efficiency of CNF material in the macroalgae matrices. However, increasing the fibre loading beyond had no significant effect on both the tensile strength and the young modulus for both modified and unmodified films, suggesting that poor interfacial bonding might have occurred between reinforced CNF and the macroalgae matrix after 4% fibre loading [29]. Incorporation of fibre beyond (>4 wt.%) resulted in the agglomeration of CNFs due to poor dispersion in the carrageenan matrices. As a result, both the tensile strength and tensile modulus of the films decreased. This indicates a lack of adhesive interaction between the reinforcement and the matrix [30].

The elongation at break (EB) exhibited a gradual steep increase for both modified and unmodified film until after 4% fibre loading. EB exhibited a similar trend compared to the tensile strength and modulus of elasticity for both modified and unmodified film. The modified films denoted high EB compared to the unmodified films as the percent fibre loading was increased, which agrees with previous studies [31]. The optimum EB was achieved at 4% fibre loading. The tensile toughness result indicated the impact of incorporating CNFs to the macroalgae matrices as the percentage fibre loading increases. However, there was a noticeable decrease in tensile toughness at 5% fibre loading, and no significant improvement of toughness was achieved. However, in all fibre loading conditions, the modified films exhibited improved tensile strength and young modulus compared to the unmodified films due to the adhesive interaction of the CNF fibres in the macroalgae matrices, which indicated improved mechanical properties. Improvement of the mechanical properties could better be attributed to the good interfacial interaction between CNFs and the carrageenan matrices and the polysaccharide structures’ influence. The enhanced mechanical strength of the silane-modified macroalgae CNF films as compared to the unmodified macroalgae CNF films might be attributed to the presence of cross-linked Si–O–Si bonds of polysiloxane [4,32].

### 3.4. Wettability of Macroalgae Kenaf Bast CNF Biopolymer Film

**Figure 4:**
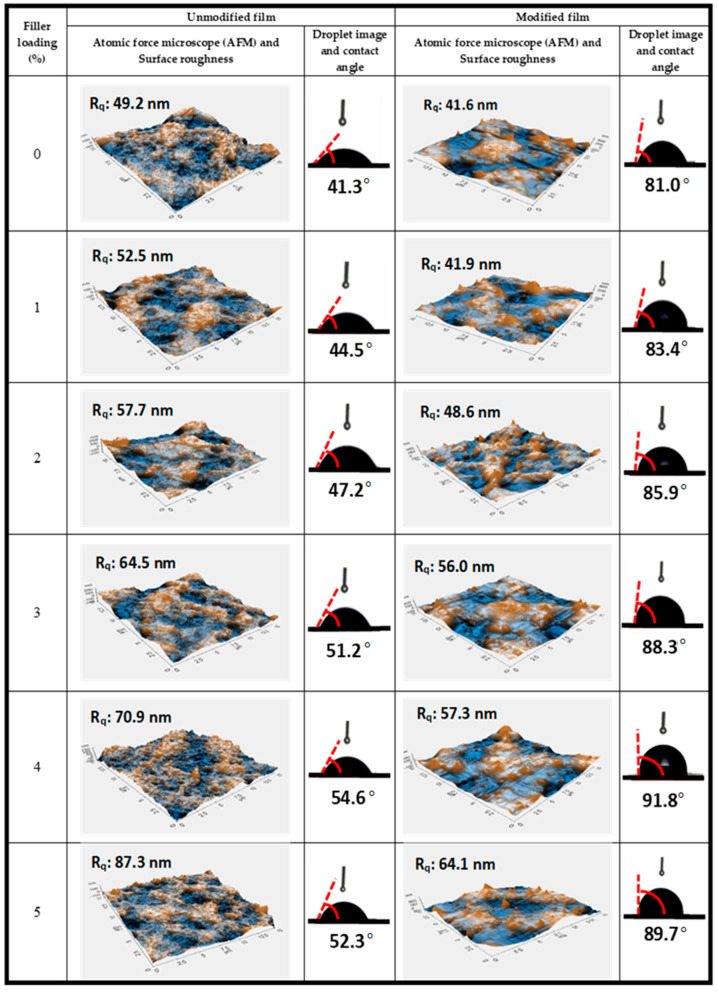
The contact angle and atomic force microscope (AFM) with a surface roughness of the unmodified and modified film.

The surface wettability and topography of the modified and unmodified macroalgae CNF biopolymer films were characterised using water contact angle (WCA) measurement and the atomic forced microscopy, as illustrated in Figure 4. The unmodified neat macroalgae films had a WCA of 41.27° and an equivalent roughness value of 49.2 nm due to their strong hydrophilic property. The hydrophobicity of the unmodified macroalgae CNF film increased with an increase in fibre loading as the surface roughness increased. The surface modification of the biopolymer films with silane resulted in a remarkable reduction in its hydrophilicity with a slight decrease in the modified film’s surface roughness for neat macroalgae and macroalgae CNF films. The increase in the fibre loading in the macroalgae matrix further improved the modified biopolymer films’ hydrophobicity. At 4% incorporation of CNF, the surface of the silane-modified biopolymer film became hydrophobic. At this fibre loading, the highest reduction in the unmodified biopolymer film’s hydrophilic property was also achieved. This could be attributed to the strong intermolecular bonding between the hydroxyl groups of the macroalgal CNF film with the silane, which reduced the moisture content due to the reduction of the free hydroxyl group biopolymer film [31]. However, beyond 4% fibre loading, the WCA increased for both modified and unmodified film due to the heterogeneous dispersion of CNF in the matrix due to unequal bonding of molecules in the biopolymer film [33]. This indicated that agglomeration of CNF, as shown in Figure 2, presumably weakened the CNF fibre interaction in the macroalgae matrix and better explained the slight decrease in the tensile strength in Figure 1. This resulted in the exposure of the film surface to the free hydroxyl group, which is attributed to the surface’s affinity to OH^−1^ group, thereby causing increase in the moisture content of the biopolymer film.

### 3.5. Thermal Behaviour of Macroalgae Biopolymer Films

**Figure 5:**
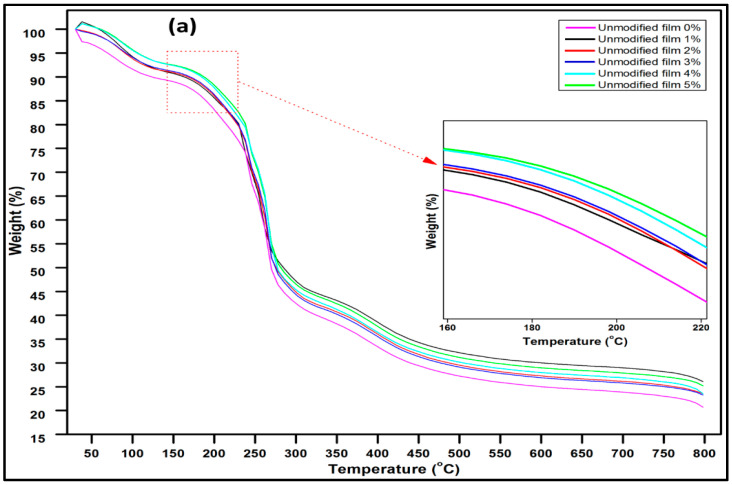

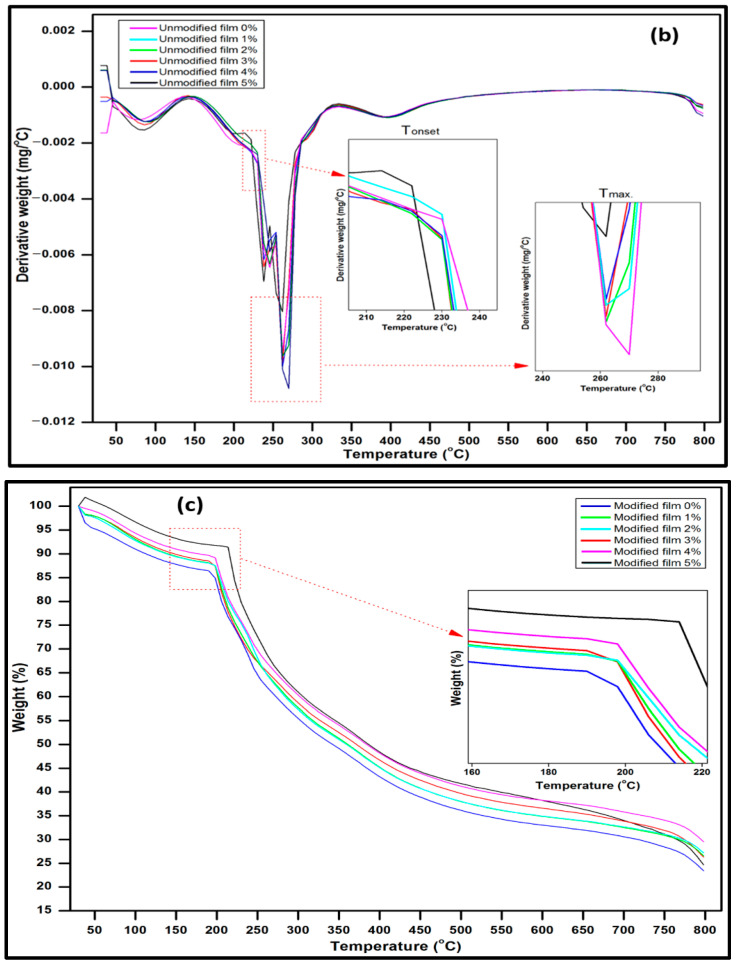

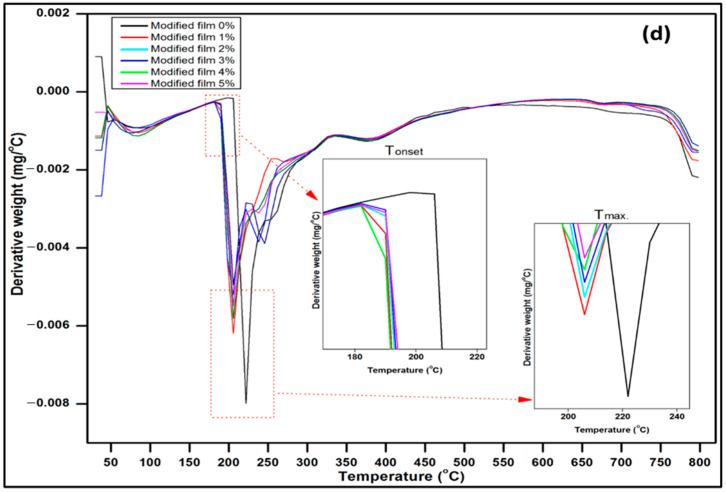
(**a**) Thermogravimetric analysis (TGA) properties of the unmodified film, (**b**) Derivative thermal gravimetric (DTG) properties of the unmodified film, (**c**) Thermogravimetric analysis (TGA) properties of the modified film (**d**) Derivative thermal gravimetric (DTG) properties of the modified film.

The thermal stabilities of the unmodified macroalgae biopolymer film and the silane-modified macroalgae biopolymer film at intervals of fibre loadings are represented by the TGA and DTG curves in Figure 5a–d. The initial weight loss occurred around 100 °C due to water evaporation. The initial decomposition occurred due to bound water molecules within the biopolymer film around 100–140 °C [34]. The DTG curve also demonstrated the dehydration at this stage. The second decomposition was achieved in the range of 220–330 °C for the unmodified film and 220 to 450 °C for the modified film. According to previous studies, degradation around 220 °C suggests that dehydration and decomposition of macroalgae and glycerol occurred in the biopolymer films [35,36]. However, approximately 49% weight loss was achieved at this stage around 280 °C for the unmodified film. There was a remarkable weight loss (60%) at this stage for the modified film. This demonstrated the impact of the silane modification of the film. The third decomposition was assigned at an elevated temperature of 340 °C, which occurred due to final degradation to ash. An increase in thermal resistance due to an increase in the decomposition temperature was achieved with the addition of CNF, as evidenced in the DTG curve.

It can be inferred that an increase in the homogenous dispersal of CNF increased the interfacial bonding with macroalgae for both the modified and unmodified film even though higher weight loss was achieved as observed in the modified film. As a result, polysaccharide macroalgae chains in the matrix were prevented from moving with an increase in temperature. Moreover, the silane-modified film achieved the highest thermal resistance at peak decomposition around 450 °C due to the effect of the matrix’s degradation and the dehydroxylation attributed to the silanol groups on the modified film, which improved surface hydrophobicity [37]. This indicated that higher thermal stability was achieved by incorporating CNF and silane modification of the films.

### 3.6. Mechanism of CNF Macroalgae Film Surface Treatment Using Triethoxymethylsilane

**Figure 6:**
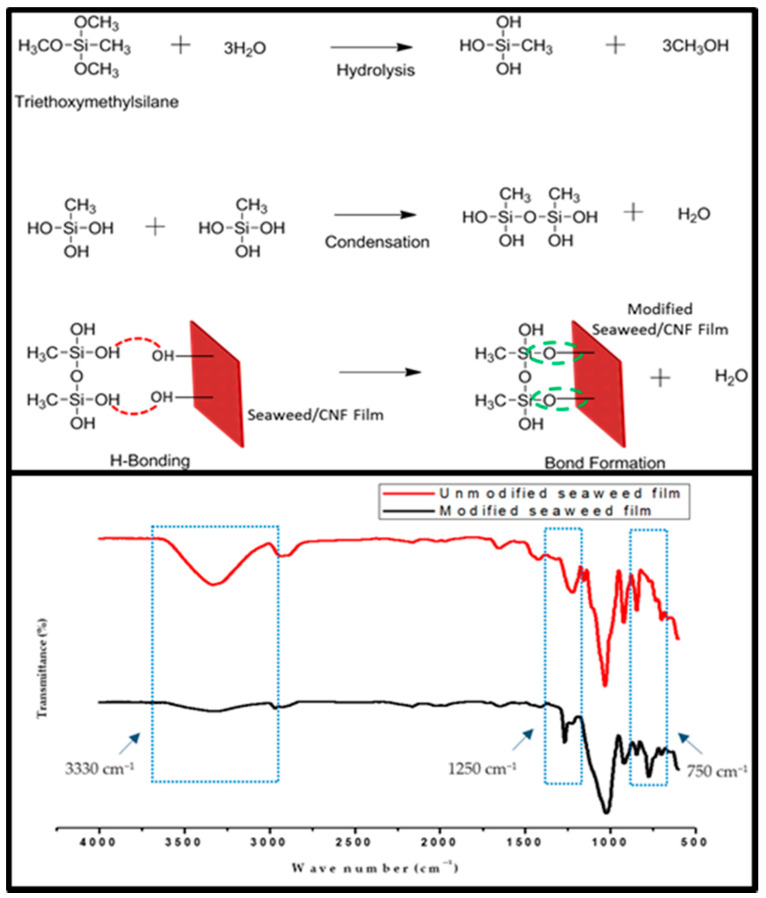
Mechanism of neat macroalgae film surface treatment using triethoxymethylsilane and FT-IR comparison of unmodified and modified neat macroalgae film.

The schematic chemical reaction mechanism and FT-IR analysis showing the silane bonding is presented in Figure 6. The chemical modification reaction of macroalgae CNF film using triethoxymethylsilane is shown in Figure 6, resulting in the formation of alkoxysilanols. During the condensation reaction, silanols were converted to siloxanes. The reaction of the silanols with the hydroxyl groups of the macroalgae CNF biofilm resulted in the hydrolysis of triethoxymethylsilane in the biofilm. As a result of the reactions, the hydroxyl groups in the macroalgae CNF biofilm were used. The hydroxide was bonded with silane to form silanols (silane coating) in the biofilm.

The functional groups’ significance in the unmodified and modified macroalgae films is illustrated in FT-IR analysis (Figure 6). A strong band assigned to both films at 3500 bands indicated the presence of OH groups. At this band, there was a noticeable reduction in the spectrum assigned to the OH group. This could be as a result of the silane bonded with the macroalgae matrix. The appearance of a strong band at 750–775 cm^−1^ represents the vibrational characteristic of CH_3_ silane. Furthermore, Si–CH_3_ bending vibration characteristic was observed at 1250 cm^−1^. The intensity of this band denoted an increase in the triethoxymethylsilane in the reaction [38]. These results suggested that the macroalgae film’s modification was very effective using silane as a coupling agent.

## 4. Conclusions

Macroalgae CNF biopolymer films were fabricated and were compared to silane-modified macroalgae CNF biopolymer film. Supercritical extracted CNF served as reinforcement for the films and were extracted from Kenaf bast fibres. The morphology, structural, mechanical, and thermal behaviour of the biofilms were investigated using the TEM, SEM, TGA, AFM, contact angle, and FT-IR analysis. The effect of the reinforcements of the fibre loadings indicated improved mechanical, thermal, and wettability properties for both modified and unmodified films with optimum fibre loading at 4%. In general, improved properties of the films were achieved as the fibre loading was increased. The macroalgae CNF biopolymer film’s wettability properties were successfully enhanced with silane treatment, which indicated that the modified film demonstrated less affinity towards water molecules. Furthermore, the silane-modified films exhibited better mechanical properties and thermal stability than the unmodified films. The improved macroalgae biopolymer films are potentially suitable materials for packaging applications.

## Data Availability

Not applicable.

## Sample Availability

Samples of the compounds are available from the authors.
